# Short treatment of metformin attenuated insulin resistance and hepatic steatosis in obese mice by mechanisms independents of PPAR-alpha

**DOI:** 10.1186/2049-3002-2-S1-P73

**Published:** 2014-05-28

**Authors:** Alexandre Teixeira, Camila Souza, Edson Lima, Helena Batatinha, José Rosa Neto

**Affiliations:** 1 Department of Cell and Developmental Biology, Biomedical Sciences Institute, São Paulo, São Paulo, Brazil

## Background

Nonalcoholic steatohepatitis (NASH) is characterized by accumulation of fat in the liver without excessive alcohol intake [1]. The optimal treatment for this disease is not well established, however, metformin is described as an efficient treatment for NASH and hepatic inflammation [2]. Based on these data, the present study aimed to evaluate the possible immune-metabolic effects of metformin in high-fat diet wild type (C57) and knockout for PPAR-alpha (KO) mice.

## Materials and methods

C57 and KO mice were submitted to a balanced or high fat diet (HFD) for 12 weeks, after 10 weeks these animals were treated with metformin or phosphate buffered saline by gavage. The insulin tolerance test (ITT) and glucose tolerance test (GTT) were performed. Histological slices of the liver, cored by hematoxilin and eosin were obtained, and the concentration of triacylglycerols and the cytokines levels were determined by ELISA.

## Results

Animals submitted to HFD showed higher gain of weight (C57 p<0.001;KO p<0.05), that in C57 was represented by a greater gain of adipose tissue (p<0.001), while the weight of the liver did not change. This diet promotes insulin resistance in C57 and KO, as observed by reduction of glycemia in ITT (p<0.05) and by an increase in GTT (p<0.001). Although no differences in liver weight were observed in KO, these animals showed higher hepatic deposition of triacylglycerols (p<0.01) and more severe steatosis without an increase of pro-inflammatory cytokines levels. After 7 days, both C57 and KO treated with metformin showed an improvement in glucose tolerance in the GTT (p<0.05), and after 10 days metformin reduced the steatosis as evidenced by histology. Controversially, the liver of C57 mice submitted to HFD and treated with metformin showed higher levels of IL1-β (p<0.05), IL-8 and IL-12 (p<0.01).

## Conclusion

The treatment with metformin improves glucose tolerance and decreases steatosis in mice submitted to high fat diet. However, the treatment was not able to reverse the inflammation in C57 mice. The mechanism of metformin is independent of PPAR-alpha.
